# Signal transduction, dimerization, and therapeutic targeting of Orexin and receptor systems

**DOI:** 10.3389/fphar.2025.1697406

**Published:** 2025-11-26

**Authors:** Shengnan Zhang, Peixiang Wang, Bingyuan Ji, Yuming Shao, Sen Hou, Jing Chen, Chunmei Wang

**Affiliations:** 1 Neurobiology Key Laboratory of Jining Medical University, Jining, China; 2 College of Forensic Medicine of Jining Medical University, Forensic Science Center of Jining Medical University, Jining, China; 3 Division of Biomedical Sciences, Warwick Medical School, University of Warwick, Coventry, United Kingdom

**Keywords:** GPCRs, GPCR signaling, neuropharmacology, neurology, depression

## Abstract

Orexin receptors (OXRs), including OX1R (HCRTR1) and OX2R (HCRTR2), are G protein-coupled receptors (GPCRs) that are activated by endogenous orexin peptides (OXA and OXB) and have potential pleiotropic effects in nervous system, which makes them highly valuable therapeutic targets. Emerging evidence indicates that OXRs exhibit a significant propensity to form homodimers and heterodimers with various GPCRs, generating biased signaling that may offer a platform for precision pharmacology and enable the design of pathway-specific drugs with fewer off-target effects. Current and emerging OXR antagonists demonstrate efficacy in sleep disorders, metabolic dysregulation, and psychiatric conditions. Furthermore, transmembrane (TM) peptides targeting specific dimer interfaces represent a novel therapeutic strategy. This review synthesizes current understanding of orexin receptor systems, focusing on the structural composition, signal transduction pathways, dimerization properties, and antagonist compounds of OXRs. We present a comprehensive overview of the current state of research, investigate the molecular pathological mechanisms associated with neurological disorders, and evaluate potential therapeutic targets for pharmaceutical development.

## 1 Introduction

Orexin receptors (OXRs) belong to the class A (rhodopsin-like) G protein-coupled receptor (GPCR) family. They are divided into two subtypes based on structure: type 1 (OX1R, also named as hypocretin receptor 1 (HCRTR1) and type 2 (OX2R, or HCRTR2). OX1R and OX2R share high sequence homology in human; 64% for the entire amino acid sequence and 80% for transmembrane regions (Kukkonen et al., 2024). The two subtypes are widely distributed in different brain regions and peripheral organs. Expression of OXRs is affected by developmental stage, energy metabolism, and disease status, and the activities of OXRs are affected by endogenous regulatory factors and dimerization (Alain et al., 2021; Smolinska et al., 2015).

Orexin, also known as hypocretin originally identified in the hypothalamus, is an endogenous ligand of OXRs and functions as a critical brain-gut signaling molecule. Orexin exists as isopeptides orexin A (OXA) and orexin B (OXB), derived from the same prepro-orexin (PPO) precursor peptide.

In the central nervous system (CNS), OXA/OXB bind to OX1R and OX2R to activate G proteins and induce specific signaling pathways, thereby regulating feeding behavior, sleep/wakefulness states, emotional behavior, and pain processing (Bigalke et al., 2022; Gotter et al., 2012). In peripheral tissues, OXA/OXB similarly bind to these receptors to modulate cardiovascular activity, gastrointestinal motility and secretion, pancreatic β cell function, and energy metabolism. (Bigalke et al., 2022; Gotter et al., 2012; Tsunematsu and Yamanaka, 2012). This dual central and peripheral action of orexins highlights their importance in coordinating multiple physiological processes throughout the body.

Our laboratory has been engaged in research on the biological functions and molecular mechanisms of orexin/receptor systems for over a decade. Our results reveal that orexin/receptor systems are associated with numerous functions. OXA can reduce cerebral ischemia-reperfusion injury (IRI) by modulating various signaling pathways, including OX1R-mediated MAPK/ERK, MAPK/p38, NF-κB, mTOR, and PI3K (Kong et al., 2019; Xu et al., 2021a; Xu et al., 2021b). These pathways are involved in regulating cell apoptosis, inflammation, autophagy, and endoplasmic reticulum stress (ERS). However, the effects of OXA on these pathways are context-dependent, and the inhibition of some pathways (e.g., PI3K and MAPK/ERK) might have inverse effects depending on the specific cellular environment. In another study on chronic unpredictable mild stress (CUMS) depression model rats, OXA was found to significantly improve the pain threshold and reduce the pain perception ability of depressed rats (Zhang R. et al., 2022).

Additionally, similar to most GPCRs, OXRs can form homodimers and heterodimers with themselves and other GPCRs, thereby performing diverse functions by enhancing or weakening signal pathways (Chen et al., 2015; Zhang R. et al., 2022). This article reviews the composition, signal transduction, dimerization, and research progress of orexins and OXRs as therapeutic targets. The article explores in detail the molecular mechanism of the orexin receptor system, its association with neurological diseases, and evaluates its potential as a target for drug development.

## 2 Features of orexin/receptor systems

Orexin/receptor systems are composed of neuropeptides OXA, OXB, and their receptors OX1R and OX2R, which are highly conserved among mammalian species (Wang et al., 2018). Conservation is not only reflected in the high similarity of amino acid sequences, but also in the stability of structures and the consistency of functions. These systems have attracted the attention in the scientific community as potential therapeutic targets for treating multiple pathological conditions.

### 2.1 Endogenous ligands OXA and OXB

OXA and OXB, two neuropeptides primarily synthesized and secreted by orexin-producing neurons located within the lateral hypothalamus (LH) and its adjacent brain regions, were independently discovered by two separate research teams in a remarkable near-simultaneous breakthrough in 1998 (De Lecea et al., 1998; Sakurai et al., 1998). The same precursor peptide PPO (131 amino acids) is hydrolyzed into two orexin subtypes in human; OXA (33 residues) and OXB (28 residues). OXA is lipophilic and has a significantly higher content in tissues and blood than OXB. (Sakurai et al., 1999).

Current evidence regarding the BBB permeability of orexins remains inconclusive. Some studies suggest that OXA’s molecular structure may confer relatively higher stability, potentially allowing a greater proportion of *peripherally-derived* OXA to penetrate the BBB via diffusion (Kastin and Akerstrom, 1999). Comparatively, when administered peripherally, OXB appears to undergo more rapid enzymatic degradation in the systemic circulation. This rapid peripheral degradation could limit the amount of OXB available to cross the BBB from the periphery into the CNS. It is important to note that under endogenous conditions, central orexin levels are predominantly determined by synthesis within neurons of the hypothalamus. Therefore, the differential BBB permeability of *peripherally administered* orexins may not directly reflect their relative concentrations measured in the CNS under physiological conditions. In fact, some studies have reported higher concentrations of OXB within the CNS (Mondal et al., 1999), underscoring the dominance of central production and regulation.

The amino acid sequences of OXA and OXB are similar, sharing 46% sequence identity across the whole sequence, but 68% in their C-terminal regions (residues 15–33). The sequence of the N-terminal region of OXA, from residues 1 to 14, includes two disulfide bonds, which is the main difference from OXB (Takai et al., 2006). Truncated OXA analogs containing two disulfide bonds are comparable in activity to native OXA. The shortest highly active OXB analog is OXB 6–28 (residues 6–28).

### 2.2 GPCRs OX1R and OX2R

Unlike the specific expression of orexin neurons in LH, OX1R and OX2R are widely distributed in brain regions and display significant differences and specificities. OX1R is mainly distributed in the LH area, ventromedial hypothalamus (VMH), hippocampus (CA), dorsal raphe (DR), locus coeruleus (LC), limbic system, and cingulate cortex, with the highest content of OX1R mRNA in VMH (Lu et al., 2000). OX2R is mainly expressed in the cerebral cortex, hypothalamus, paraventricular nucleus (PVN), and preoptic nucleus. The PVN mainly expresses OX2R, while monoaminergic neurons in the papillary tuberosity nucleus only express OX2R. There is partial overlap in the distribution of OX1R and OX2R, and simultaneous expression in areas such as the hippocampus, anterior hypothalamus, amygdala, DR, pontine tegmental nucleus (PPT), and dorsal lateral tegmental nucleus (LDT) (Lu et al., 2000). A new *in situ* hybridization chain reaction technique was recently developed to observe the cerebral regions of OXR subtypes in mouse (Tsuneoka and Funato, 2024). In the brain, only a few cells from specific brain regions have been found to express both OX1R and OX2R simultaneously, such as the DRN and the VMH. In many cerebral regions, expressions of OX1R and OX2R vary in different cell types. Moreover, most orexin neurons were not found to simultaneously express OXRs.

OXRs are also widely present in peripheral organs such as the gastrointestinal tract, pancreas, and adrenal gland. OXRs exhibit distinct distribution patterns within the gastrointestinal tract, with OX1R predominantly localized in gastric fundus enterochromaffin-like cells, while OX2R shows higher expression density in myenteric plexus neurons of the small intestine (Noorani et al., 2021). Notably, OX1R is highly expressed in the affected colonic epithelium of most ulcerative colitis patients, but not in the non-affected colonic mucosa, suggesting region-specific regulatory mechanisms (Messal et al., 2018). By means of RT-PCR, it was found that both OXR1 and OXR2 are expressed in rat pancreatic islets. Furthermore, the expression levels of OXR1 were higher than OXR2 (Nowak et al., 2005). This cellular specificity suggests differential regulatory roles of orexin signaling in glucose homeostasis. OX1R is predominantly expressed in zona glomerulosa of the adrenal cortex, while OX2R shows higher density in the adrenal medulla (Blanco et al., 2002). OXA and OXB combine OX1R or OX2R with different affinities. OXA preferentially binds to OX1R, with an affinity 5 to 100-fold higher than OXB, whereas both OXA and OXB have almost equal binding affinity with OX2R, leading to speculation that OX1R is a specific receptor for OXA, while OX2R is a non-selective receptor for OXA and OXB (Ammoun et al., 2003). In addition, OXA 2–33 (residues 2–33) was found to bind preferentially to OX1R, but OXB 10–28, and OXB 6–28 have highly potent selectivity for OX2R (>1000-fold) (Lang et al., 2004).

### 2.3 Functions of orexin/receptor systems

OX1R and OX2R exhibit differential but complementary distributions, and combine ligands with different affinities, suggesting that they have overlapping but different physiological functions and potential therapeutic profiles. Initial research found that orexin/receptor systems play fundamental roles in regulating feeding behavior, sleep and wakefulness, narcolepsy and insomnia (Panda et al., 2025), reward circuits, drug addiction, and spatial cognition (Haghparast et al., 2017; Inutsuka and Yamanaka, 2013; Yamanaka et al., 1999; Zhou et al., 2011). Especially in ventral tegmental area (VTA), after activation, OXRs can enhance the excitability of VTA dopaminergic neurons, which is closely related to reward seeking behavior in animals, and can also regulate the response of VTA dopaminergic neurons to drug stimulation.

In the peripheral nervous system, orexins have been shown to exert a variety of effects, such as, increasing heart rate and blood pressure (Gao et al., 2021), modulating gastrointestinal motility and secretion (Bulbul et al., 2010), promoting lipolysis and thermogenesis (Liu et al., 2020).

**FIGURE 1:**
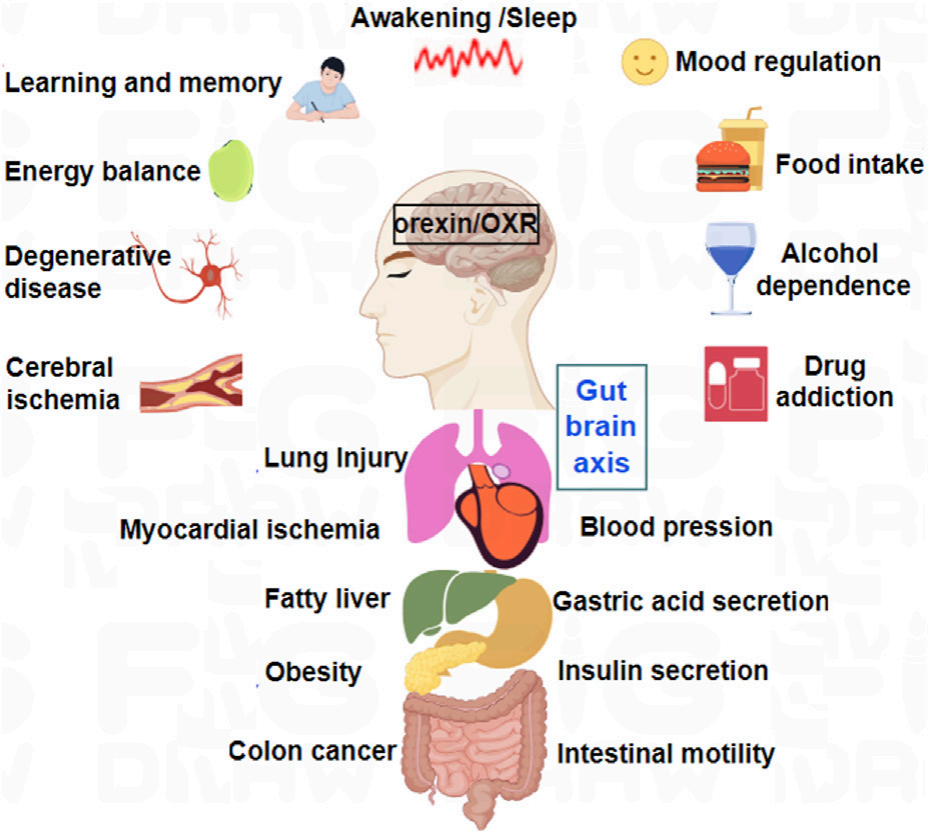
Major physiological functions of orexins and their receptors in CNS and peripheral nervous system (PNS).

Later, orexin/receptor systems were found to have positive effects on regulating emotional behavior, recovering body temperature, and reducing oxidative stress responses (Rotondo et al., 2023; Soya and Sakurai, 2020; Sun et al., 2023). In recent years, the pathophysiological roles and mechanisms of orexins and their receptors in neurological diseases, such as Alzheimer’s Disease (AD), Parkinson’s Disease, epilepsy, depression, hypersomnia, and cognitive impairment, have also attracted increasing attention (Carpi et al., 2024; Lu et al., 2025; Ten-Blanco et al., 2023) (Figure 1). Furthermore, numerous research groups have extensively explored the roles of orexins in various peripheral pathologies, including chronic inflammation (Messal et al., 2018), pancreatitis (Sassek et al., 2017), and colon cancer (Wen et al., 2022).

In summary, the role and significance of the orexin/receptor system in pathology far exceed current research and understanding. Its numerous roles in the central and peripheral nervous systems highlight its potential as a therapeutic target for various diseases.

## 3 Signaling pathways mediated by OXRs

Following the canonical GPCR model, active OX1R and OX2R transduce extracellular signals via conformational changes. This triggers two primary signaling branches: the activation of heterotrimeric G proteins, which mediates G protein-dependent signaling, and the recruitment of β-arrestin, which is responsible for β-arrestin-dependent (G protein-independent) signaling.

**FIGURE 2:**
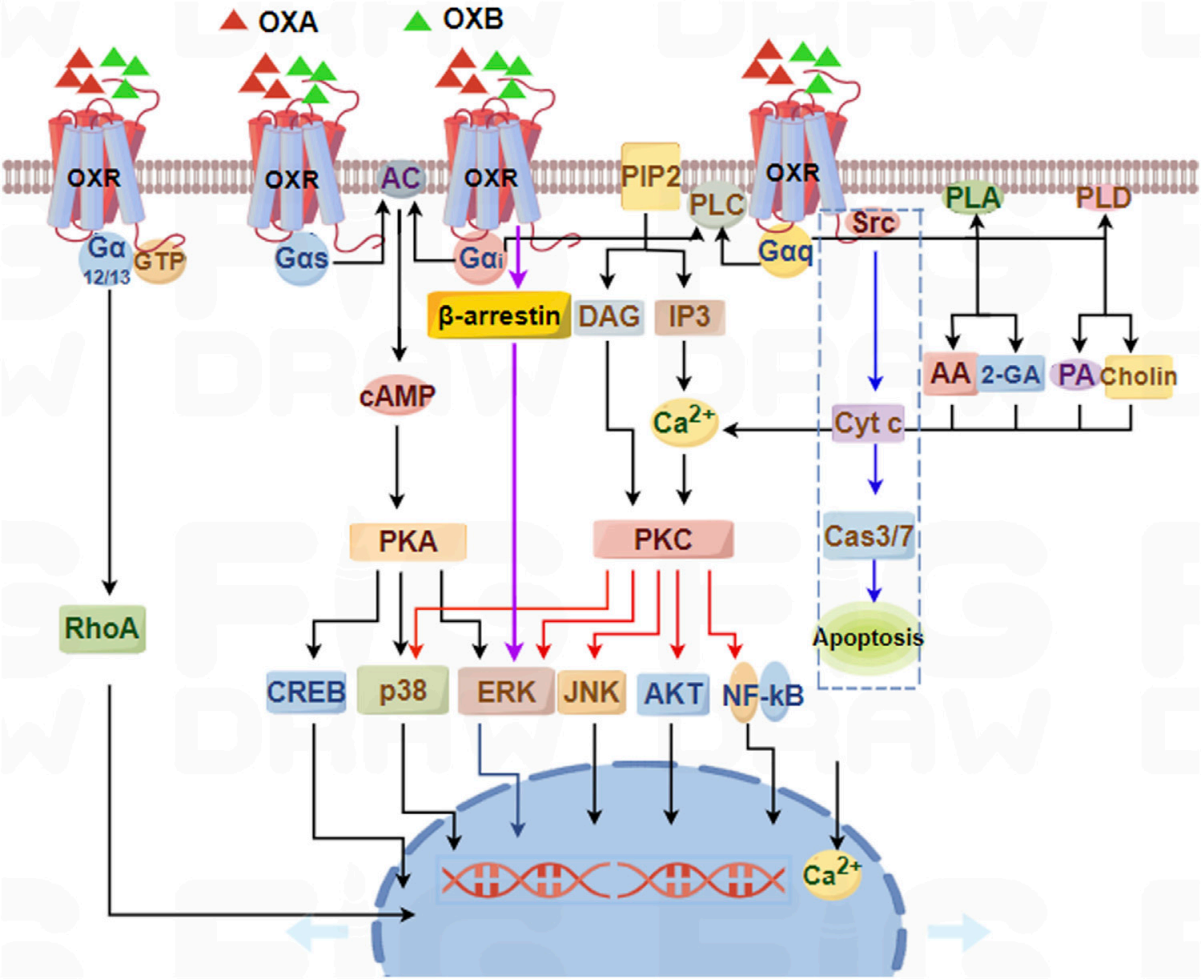
Schematic summary of signaling pathways mediated by orexin/receptor systems. OX1R and OX2R can couple to Gα_q_, Gα_s_, Gα_i/o_, and Gα_12/13_ subtypes, which subsequently induce PLC, PLA, PLD, or AC, ultimately resulting in an increase in cytosolic [Ca^2+^] and a downstream cascade response through Gα_q_-PLC-PKC or Gα_s_/Gα_i_-AC-PKA pathways.

OXA/OXB first bind OX1R/OX2R, which activates G-protein isoforms (Gα_q_, Gα_s_, Gα_i/o_, and Gα_12/13_) or recruit β-arrestin (Kukkonen et al., 2024). These effectors promote the activities of phospholipases, protein kinases, and ion channels, subsequently inhibiting or enhancing downstream different signaling pathways, and finally exerting multiple biological effects in different systems (Figure 2).

Due to the specific expression OX1R and OX2R, and the unequal affinities of OXA and OXB for OX1R and OX2R, it is obvious that the specific signaling pathways are relatively diverse and complex, and cell-, tissue-, and cellular context-dependent. A relatively comprehensive signaling pathway map was derived for OXA/OXB-stimulated reactions (Chatterjee et al., 2023). This map contains 318 downstream molecules classified into five categories: 226 gene regulation, 31 catalysis, 23 activation/inhibition, 23 transport, and 15 molecular association. The map is a useful resource for further analysis of signaling mediated by orexins (Chatterjee et al., 2023).

### 3.1 Heterotrimeric G proteins

The human genome encodes 16 Gα genes divided into four subfamilies: Gα_q_, Gα_s_, Gα_i/o_, and Gα_12/13_. Following OXR activation, Gα_q_ was initially thought to be coupled and subsequently induce downstream signaling to evoke complex cellular responses. Latter research further expanded the partners of the G-protein subtypes coupled with OXRs, with varying degrees of coupling to different G proteins (Gα_q_, Gα_s_, Gα_i/o_, and Gα_12/13_) depending on ligand, cellular environment, and experimental method (Kukkonen and Turunen, 2021).

Heterotrimeric G proteins coupled with OX1R or OX2R have been directly identified in only a few primary studies. Photoaffinity labeling revealed that OXA binds OX2R to activate Gα_q_, Gα_s_, and, to a lesser degree, Gα_i_, but not Gα_o_, in adult adrenals only expressing OX2R, but not OX1R (Randeva et al., 2001). OX2R has the ability to activate Gα_q_, Gα_i_, and Gα_s_ using [^32/33^P] GTP-azidoanilide, and OXRs couple to Gα_i/o_ using [35S] GTPγS (Bernard et al., 2002; 2003). They found that OX1R and OX2R levels were significantly elevated in the hypothalamus of food-deprived animals. Interestingly, food deprivation decreased adrenal OX1R and OX2R levels. In the hypothalamus of food-deprived rats, coupling of orexin receptors to Gα_q_, Gα_s_, and Gα_o_ was significantly enhanced, but coupling to Gα_i_ was decreased. Meanwhile, in adrenal cortex there was less coupling of OX1R and OX2R to Gα_q_, Gα_s_, and Gα_o_, and enhanced coupling to Gα_i_. It was implied that food deprivation has opposite effects on the expression and signaling of OX1R and OX2R in hypothalamus and adrenal cortex (Karteris et al., 2005). In the hypothalamus, OX1R is mainly involved in regulating physiological functions such as wakefulness and appetite. OXB can stimulate the sympathetic nervous system by activating OX1R, thereby affecting the cardiovascular system (Herbenick et al., 2019). In the adrenal cortex, activation of OX1R may promote the secretion of adrenal cortical hormones, which can further affect the function of the cardiovascular system (Imperatore et al., 2017).

However, most subsequent studies indirectly deduced that coupling of receptors to G-protein subtypes relied on the impact of receptor activation on downstream signals via *in vitro* experiments. Early work showed that OX2R mainly activates the Gα_q_ subtype based on receptor coupling to increase [Ca^2+^], and later findings revealed strong activation of phospholipase C (PLC) in various recombinant cell lines (Holmqvist et al., 2002; Lund et al., 2000). Results from cell lines stably expressing OX1R or OX2R indicated that both receptors coupled to Gα_q_, but OX2R and not OX1R coupled to Gα_i_, and neither receptor coupled to Gα_s_ (Zhu et al., 2003). Based on robust and concentration-dependent [Ca^2+^] elevation, and cAMP and IP_3_ production in Chinese hamster ovary (CHO) cells stably expressing OX1R, OX1R couples to a minimum of 3 G proteins (Gα_i/o_, Gα_s_, and Gα_q_) in CHO cells (Holmqvist et al., 2005). In H295R adrenocortical cells, ERK_1/2_ and p38 were observed to undergo rapid phosphorylation in response to OXA and OXB, predominantly mediated by Gα_q_ and, to a lesser extent, Gα_s_, while phosphorylated p38 also exhibited a minor Gα_i_-dependent component (Ramanjaneya et al., 2009). However, this observation is based on a single study and may not be universally applicable to other cell types or contexts.

Based on present *in vitro* and *in vivo* results, in recombinant cell lines except for Gα_12/13_ which is not assessed, OX1R and OX2R readily couple to Gα_q_, Gα_s_, and Gα_i/o_ families. In endogenous cells, OX1R and OX2R are probably capable of coupling to all these G-protein families, but their combined stage may be subject to regulation at the tissue, species, and environment level. These differences in G proteins activated by OX1R and OX2R can also be attributed to the different ligands used for stimulating receptors.

Specifically, the primary G proteins associated with OX1R and OX2R activation are, and Gα_q_ and Gα_s_, which mediate downstream signaling pathways such as the activation of PLCC and AC, respectively. Additionally, the promiscuous nature of orexin receptors, explaining that they can also bind to other G protein isoforms, including Gα_i/o_ and Gα_12/13_, depending on the tissue context. This promiscuity allows orexin receptors to modulate diverse physiological functions through different signaling pathways.

### 3.2 PLC pathway

Upon activation, Gα_q_ triggers membrane-associated PLC to PIP_2_ into IP_3_ and DAG. IP_3_ diffuses to the endoplasmic reticulum (ER) where it binds to specific ligand-gated Ca^2+^ release channels (IP_3_ receptors), inducing calcium efflux from ER stores into the cytosol. The concomitant elevation of cytosolic Ca^2+^ synergizes with membrane-bound DAG and phosphatidylserine to activate conventional protein kinase C (cPKC) isoforms. Activated PKC then phosphorylates components of the MAPK/ERK cascade, initiating downstream signaling events that regulate diverse cellular processes including proliferation, differentiation, and apoptosis control (Littmann et al., 2021; Rebres et al., 2011).

The Gα_q_-PLC-PKC pathway is an important signaling pathway for OXRs in recombinant cells. Orexins promote catecholamine secretion from human pheochromocytomas, acting through the OX2R-PLC-PKC signaling pathway (Mazzocchi et al., 2001). Using whole-cell patch-clamp techniques, the excitatory roles of OXA on nucleus tractus solitarius neurons were found to be mediated through activating PLC-PKC-NSCC signaling pathways (Yang et al., 2003).

Binding orexins to OXRs can cause a significant increase in [Ca^2+^] that is mainly triggered by activating PKC-dependent pathways. After OXA stimulation by OX1R, the [Ca^2+^] in rat neuropeptide Y (NPY) neurons is significantly increased in a Gα_q_-PLC-IP_3_-PKC-dependent manner (Muroya et al., 2004). OXA binding to OX1R increases [Ca^2+^] in prefrontal cortex neurons, which depends on the influx of [Ca^2+^]e caused by activating L-type channels through the PLC-PKC signaling pathway (Xia et al., 2009). In the arcuate nucleus of the hypothalamus, OXA upregulates voltage-gated L-type calcium channels through OX1R-PLC-PKC signaling pathway, thereby activating the hypothalamic AMPK signaling pathway (Wu et al., 2013). OXA appeared to prompt presynaptic glutamate release by activating L-type Ca^2+^ channels in bipolar cells, mediated by an OX1R-PI-PLC-PKC signaling pathway (Ruan et al., 2021).

OXA/OXB stimulation of OX1R and OXB stimulation of OX2R activates the Gα_q_-mediated PLC/PKC cascade, ultimately leading to phosphorylation of the ERK pathway. Shu et al. (2014) suggested that OXA can stimulate ERK_1/2_ phosphorylation, then promote the migration of astrocytes mainly through the OX1R-PLC-PKCα signal pathway, but not through OX2R (Shu et al., 2014). The Gα_q_-PLC pathway may also, under certain circumstances, proceed through DAG lipase. OXA may induce antinociception by activating postsynaptic OX1R (Ho et al., 2011), possibly through endocannabinoid 2-AG synthesis via the Gα_q_-PLC-DAGLα enzymatic cascade, culminating in inhibition of GABA release in the vlPAG.

### 3.3 Protein kinase A (PKA) pathway

Activated Gα_s_/Gα_i/o_ modulates the activity of AC on the cell membrane, activating or inhibiting the conversion of intracellular ATP to cAMP. As an intracellular second messenger, cAMP regulates cAMP-dependent PKA activity, subsequently controlling various biological effects through phosphorylation or dephosphorylation (Garriz et al., 2023; Stefan et al., 2011).

Orexins can suppress catecholamine release and synthesis, and the inhibitory effect is mediated, at least in part, by the OX2R-cAMP-PKA signaling pathway (Mazzocchi et al., 2001). Furthermore, OXA, but not OXB, enhanced cAMP production but did not alter IP_3_ release, exclusively acting through the OX1R-AC-PKA-dependent signaling cascade (Ziolkowska et al., 2005). Comprehensive signaling of recombinant OX2R receptor in HEK-293 cells showed that p-ERK_1/2_ induced by OXA could be mediated by Gα_q_-PLC-PKC, Gα_s_-AC-cAMP-PKA, and Gα_i_ cascades, but Gα_q_-PLC-PKC and PKA pathways were not required for p38 MAPK signaling activated by OX2R (Tang et al., 2008). In addition, orexin binding to OX2R increased postsynaptic [Ca^2+^], which was induced by activation of voltage-gated R- and T-type Ca^2+^ channels mediated by the AC-PKA pathway (Nakamura et al., 2010).

The ability of OXR to activate both stimulatory (Gα_s_) and inhibitory (Gα_i_) G-proteins is not a signaling contradiction but rather a sophisticated mechanism for fine-tuning cellular responses. This dual coupling enables dynamic regulation of cAMP, where simultaneous activation can create localized, transient cAMP microdomains rather than a futile cycle. This signaling flexibility is critically dependent on cellular context. The preferential coupling to Gα_s_ or Gα_i_ in a tissue is dictated by the relative expression levels of G-protein subunits, the presence of regulatory proteins, and receptor localization within specific membrane microdomains. Crucially, this promiscuity is not common to all GPCRs, underscoring its functional importance for the orexin system. Supporting this, Hauser et al., systematically profiled G-protein coupling and found that promiscuous coupling to all major G-protein families (Gα_s_, Gα_i_, Gα_q_) is quite rare among Class A GPCRs (Hauser et al., 2022). Their research identified the orexin OX2R as one of the few prominent exceptions, demonstrating significant promiscuity.

### 3.4 Receptor-gated ion channel pathway

Activated Gα can lead to the opening of inward rectifying K^+^(GIRKs) channels or inhibition of voltage-gated Ca^2+^ channels, and Gβ and Gγ are also involved in the activation of GIRK channels. They can regulate the ion concentration inside and outside the cell, thereby affecting the excitability and electrophysiological activity of the cell.

OXRs can elicit neuronal excitation by inhibiting phosphorylation of inward rectifier K^+^ channels GIRK and KirNB via a PTX-insensitive G protein (i.e., Gα_q_) (Hoang et al., 2004). OX1R activated a Ca^2+^ entry pathway involving diacylglycerol-activated transient receptor potential canonical (TRPC) channels in differentiated neuroblastoma cells (Nasman et al., 2006). In HEK293 cells, OXA acting at OX1R triggered oscillatory Ca^2+^ responses (Peltonen et al., 2009). These responses were attenuated by interference with the TRPC3 channel, elevated [Mg^2+^], or inhibition of phospholipase A2, suggesting calcium oscillations mediate OX1R-dependent on TRPC3 channel. Another experiment conducted on rat INS-1E cells uncovered a TRPV calcium channel-mediated Ca^2+^ influx after OX1R stimulation, leading to an increase in insulin secretion and cell proliferation through an ERK_1/2_-dependent pathway (Skrzypski et al., 2016).

### 3.5 β-arrestin signaling

In addition to coupling with classic heterotrimeric G proteins, OXRs interact with GPCR kinases (GRK2 and GRK5) and recruit β-arrestin proteins. β-arrestin binding primarily acts to desensitize the receptor, mediating internalization and terminating G-protein signaling. Furthermore, β-arrestin can serve as a scaffold to initiate independent signaling cascades through β-arrestin-dependent pathways (Cai et al., 2020; Gratio et al., 2025), leading to distinct cellular outcomes.

OXRs can bind to β-arrestin 1 and 2, accompanied by continuous stimulation with high concentrations of agonists. An observed increase in ERK_1/2_ phosphorylation at 90 min compared with 2 min was mediated by β-arrestin and G protein, respectively (Dalrymple et al., 2011). OX1R interacts with β-arrestin 2 in an agonist-dependent manner (Milasta et al., 2005), and C-terminal mutants of OX1R inhibited the phosphorylation of ERK_1/2_, and reduced β-arrestin 2 recruitments induced by agonist. Using three C-terminal mutants, which were truncated at OX2R residues 368, 384, and 414, it was demonstrated that residues 385–414 significantly influenced agonist-induced internalization following stimulation with OXA or OXB after transfection with β-arrestin 1 (Wang et al., 2017). Together, these studies have provided a deeper understanding of the role of β-arrestin in orexin/receptor signaling transduction, and confirm the potential significance of non-G protein interactions.

### 3.6 Other GPCR-interacting proteins

Besides G proteins and β-arrestin, OXRs reportedly interact with SHP-2, Tctex-type 1 and 3 (Dynlt1, Dynlt3), and Melanocortin Receptor Accessory Protein 2 (MRAP2. Orexin can induce apoptosis via OX1R, mediated by SHP-2 phosphatase recruitment (Voisin et al., 2008). Duguay et al. (2011) confirmed interactions between OX1R and dynein light chains of Dynlt1 and Dynlt3 (Duguay et al., 2011; Johansson et al., 2008). Especially, Dyntl1 seems to modulate the intracellular localization of OXR. In HEK293 cells expressing OX1R, OXA induced a less sustained ERK_1/2_ activation when Dynlt1 was co-expressed, but it was prolonged under reduced Dynlt1 expression, suggesting that Dynlt1 modulated orexin signaling by regulating OX1R. Using Co-IP and BiFC, specific interactions between MRAP2 and OX1R were confirmed (Rouault, et al., 2017). When co-expressed with MRAP2, total expression of OX1R was decreased by 30% and retained in intracellular compartments. A high cAMP response elicited by OXA was also obviously blocked by MRAP2.

### 3.7 Other orexin/receptor signaling pathways

In addition to the regular and classical signaling pathways described above, other signaling pathways have been reported, involving activation of phospholipase A2, phospholipase D, and other proteins. These further highlight the diverse signaling capabilities of orexin/receptor systems, consistent with their complex physiological functions.

Low concentrations of OXA activated PLD activity, but PLC was activated when the concentration of OXA was increased by 10–100 times (Johansson et al., 2008). In HEK293 and Neuro-2a cells expressing OX1R, OXA stimulation of OX1R can simultaneously release 2-AG and AA (Turunen et al., 2012). PLA2 was responsible for some AA releases, while DAG lipase was responsible for all 2-AG releases and some AA releases. Additionally, orexin stimulation strongly activated PLD1 in CHO cells stably expressing OX1R, which was mediated by PKCδ but not by other PKC isoforms (Jantti et al., 2012). Forte et al. found that OXA induces DAGLα synthesis of 2-AG through OX1R and then converts 2-AG to 2-AGP in primary hippocampal neurons. They also recorded through patch clamp that 2-AGP mediated phosphorylation of pT231 Tau impairs glutamatergic transmission in the mouse hippocampus (Forte et al., 2022). Morello et al. report that OXA/OX1R signaling at POMC neurons promotes 2-AG biosynthesis, hyperphagia, and weight gain by blunting α-MSH production via CB1R-induced and ERK_1/2_ activation- and STAT3 inhibition-mediated suppression of Pomc gene transcription (Morello et al., 2016). OXA significantly alleviated neuronal apoptosis following ICH by upregulating p-mTOR and p62, while it downregulated p-ERK_1/2_ and LC3 (Zhang D. et al., 2022). OX1R-silenced GCs displayed low expression of Bcl-2 and PCNA, high expression of Bax, caspase-3, TNF-α, and P21, and considerable downregulation of p-AKT and p-ERK_1/2_ (Safdar et al., 2021).

On the other hand, a new mechanism revealed that OX1R mediated apoptosis in human colon cancer cells via activation of OX1R that directly drove apoptosis through Gα_q_, independent of the classical Gα_q_ activation of PLC (Laburthe and Voisin, 2012). Activated OX1R promoted the dissociation of Gα_q_ into αq and βγ dimers. The released βγ dimers interacted with Src, phosphorylating two immunoreceptor tyrosine-based inhibitory motifs (ITIMs) of OX1R, and further activating SHP-2, thereby causing the release of cytochrome c from mitochondria and activating CASP3 and CASP7, ultimately leading to cell apoptosis (Figure 2: blue dashed box).

The research on the orexin receptor signaling pathway has benefited from the advancement of experimental methods, but these methods also face challenges in interpreting the results and establishing physiological correlations. The recent comprehensive analysis by Hauser et al. (2022) highlights the importance of understanding the selectivity of GPCR-G protein coupling through a systematic approach (Hauser et al., 2022). Their meta-analysis of the three main datasets revealed significant differences in G protein conjugation reported in different studies, emphasizing the necessity of critical evaluation of experimental methods and results.

## 4 Dimerization of OXRs

While most GPCR dimerization/oligomerization studies have been conducted in recombinant systems, emerging evidence suggests OXRs may form homodimers, heterodimers, or higher-order oligomers under physiological conditions (Thompson et al., 2017; Xu et al., 2011). Notably, OX1R has been shown to preferentially form homodimers in native brain tissue. Some heterodimerization partners have also been identified in physiological contexts. However, it should be emphasized that most current dimerization models are still based on recombinant expression systems, and the functional significance of these interactions *in vivo* requires further validation.

### 4.1 Homodimerization of OXRs

Homologous dimerization refers to mutual binding between two identical receptors or receptor subtypes. The two subunits in homodimers are usually identical, while they may exhibit unique functional properties that a single subunit may not possess.

By detecting bioluminescence resonance energy transfer (BRET) signals, OXR subtypes were found to readily form constitutive homodimers, and BRET efficiency was higher for homodimers than for almost all heterodimers (Jantti et al., 2014). OX1R exists predominantly as a homodimer at a range of expression levels in the basal state (Xu et al., 2011). This was the first study to determine the proportion of OXR present in cells as a homodimer. The agonist OXA markedly increased the proportion of higher-order OX1R complexes, while selective OX1R antagonists effectively enhanced the proportion of OX1R migrating in Blue Native-PAGE as a monomer in a concentration-dependent manner, consistent with ligand-induced reorganization of the homodimer.

### 4.2 Heterodimerization of OXRs

For many GPCRs, heteromerization can result in novel signaling distinct from the original monomers, and this applies to OXRs. OXRs, especially OX1R, have a significant propensity to form heterodimeric complexes with a variety of receptors including kappa opioid receptor (KOR), cannabinoid receptor 1 (CB1R), apelin receptor (APJ), growth hormone secretagogue receptor 1a (GHSR1a), cholecystokinin A receptor (CCK1R), 5-hydroxytryptamine 1A receptor (5-HT1AR), and G protein-coupled receptor 103 (GPR103).

#### 4.2.1 OX1R and KOR heteromerization

KOR is a GPCR widely expressed throughout CNS. KOR is specifically activated by endogenous ligand dynorphin (Dyn), and subsequently couples with Gα_i_ for intracellular signal transduction, which mainly affects AC, K^+^/Ca^2+^channel activity, PLC, and the MAPK pathway, thereby regulating pain, depression, movement, emotion, and drug addiction (Karkhanis et al., 2017; Limoges et al., 2022). OXA and Dyn are co-expressed and co-released in hypothalamic neurons, modulating brain reward mechanisms and hedonic intake in an opposing manner in VTA, which contains both OX1R and KOR (Anderson et al., 2018; Mattar et al., 2022). These findings support the hypothesis that OX1R and KOR may coordinate their functions through interactions to coordinate the release of peptides in the hypothalamus and dopamine reward system.

**FIGURE 3:**
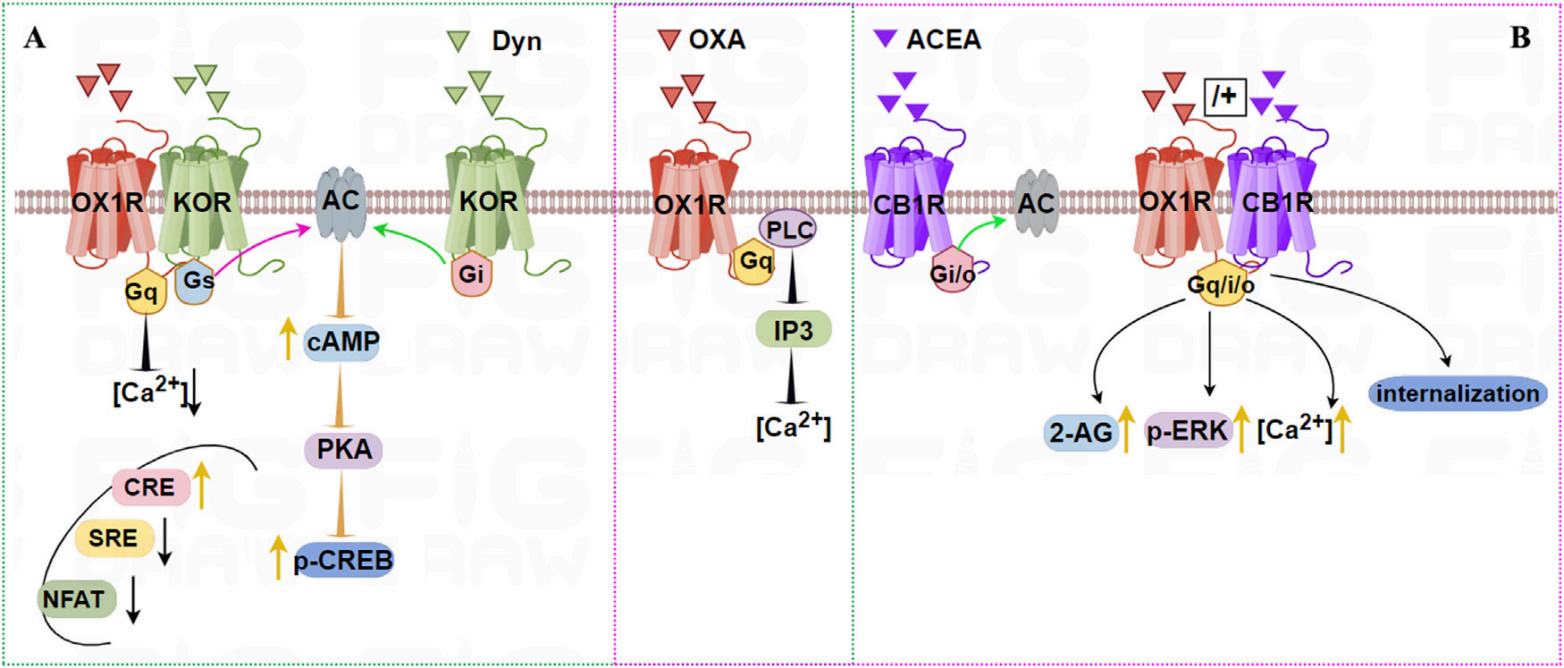
Illustration of the signaling pathways induced by dimerization of OX1R with KOR **(A)** or CB1R **(B)**. A (green dashed): OX1R with KOR can form constitutive heterodimers. Stimulation by OXA and/or Dyn activates Gα_s_, which promotes cAMP-CREB signaling. OXA and/or DynA lower the intracellular [Ca^2+^] content in OX1R/KOR cells relative to OX1R cells. CRE is increased, but SRE and NFAT luciferase activity are decreased after OX1R and KOR dimerization. B (pink dashed): OX1R forms heterodimers with CB1R following incubation with OXA, or OXA + ACEA, but not ACEA. Treatment with OXA leads to stronger and longer-lasting elevation of intracellular [Ca^2+^], 2-AG biosynthesis, and eventually to stronger activation of p-ERK_1/2_ (Thr202/185).

Chen et al. (2015) used single-cell PCR to demonstrate OX1R and KOR co-expression in rat hippocampal neurons, revealing constitutive heterodimers using BRET and fluorescence resonance energy transfer (FRET) technologies (Chen et al., 2015). After stimulation with OXA or DynA (1–13), cAMP levels were significantly higher in a dose-dependent manner in OX1R/KOR cells than in OX1R or KOR cells, while the [Ca^2+^] content in OX1R/KOR cells was lower than in OX1R cells stimulated by OXA. Additionally, compared with single transfected cells, the activity of cAMP-response element (CRE) was increased, but serum response element (SRE) and nuclear factor of activated T-cells (NFAT) luciferase activity were decreased in cells stably expressing OX1R and KOR. Furthermore, CREB activity was significantly higher in stable OX1R/KOR cell lines than in cells expressing OX1R or KOR alone (Figure 3A). These results suggest that OX1R forms a heterodimer with KOR and that induces a novel protein kinase A/cAMP-response element binding protein signal transduction. Dimerization of OX1R and KOR may inhibit the Gα_q_/Gα_i_ signaling pathways but activate the Gα_s_ signaling pathway.

#### 4.2.2 OX1R and CB1R heterodimerization

The distribution of OX1R overlaps with that of CB1R in the cerebral cortex, thalamus, amygdala, DRN, hippocampus, and LH. The physiological functions of orexins and endocannabinoids overlap in the central CNS, and are associated with sleep, appetite, and reward processing. OX1R and CB1R have a cross-activation effect (Hilairet et al., 2003). When both receptors are expressed simultaneously, CB1 enhances the activity of orexin-mediated mitotic activation protein kinase, and specific CB1R antagonists can block the above response. Kim et al. (2021) observed co-localization of OX1R-CB1R in the ventral striatum in male and female C57BL/6 mice. Combined treatment with a dual orexin receptor antagonist (DORA) and the CB1R agonist CP55940 led to increased OX1R-CB1R co-localization (Kim et al., 2021). These results demonstrate the capacity of CB1R and OX1R to interact directly with mutual regulatory characteristics.

Single-cell FRET imaging showed that OX1R and CB1R were co-expressed and present as heterodimers in intracellular vesicles (Ellis et al., 2006). Treating cell co-expressing the two receptors with SR-141716A (CB1R antagonist) or SB-674042 (OX1R antagonist) led to the two receptors relocating to the cell membrane. OX1R forms a heterodimer with CB1R, increasing signal transmission from OX1R to the ERK pathway and affecting OX1R activation, transport, and internalization (Jantti et al., 2014; Jantti et al., 2013). In hypothalamic neurons of embryonic mice, constitutive OX1R/CB1R heterodimers can form after incubation with OXA, or OXA + ACEA, but not ACEA, supporting the working of these heterodimers *in vivo* (Imperatore et al., 2016). Treatment with OXA led to stronger and longer-lasting elevation of [Ca^2+^], as well as 2-AG biosynthesis, and ultimately stronger activation of p-ERK_1/2_ (Thr202/185), as shown in Figure 3B.

Early evidence indicated that heterodimerization between OX1R and CB1R strongly enhances OX1R signaling, but some studies indicate that signal potentiation may more likely depend on the capacity of OX1R signaling to trigger CB1R ligands, thus explaining the co-signaling of OX1R and CB1R.

#### 4.2.3 OX1R and APJ heteromerization

In 1998, the endogenous peptide apelin was isolated from bovine stomach extracts and identified as the natural ligand for APJ. In the brain, APJ are mainly distributed at the PVN of the thalamus, the hippocampus, and supraoptic nucleus (SON) of the hypothalamus (O'Carroll et al., 2013). The endogenous ligands of APJ are Apelin’s (apelin-13, apelin-17, and apelin-36), and the subsequently discovered elabela, among which apelin-13 is widely used and has stronger biological activity. The apelin/elabela-APJ system plays a role in protecting against IRI, excitotoxic injury, oxidative stress damage and inhibition of neuronal apoptosis (Yan et al., 2015; Yang et al., 2015). Considering the partially overlapping distribution and the comparable roles in the brain, the apelin/elabela-APJ system interact with the orexin/OXR system in terms of regulating specific cerebral functions.

**FIGURE 4:**
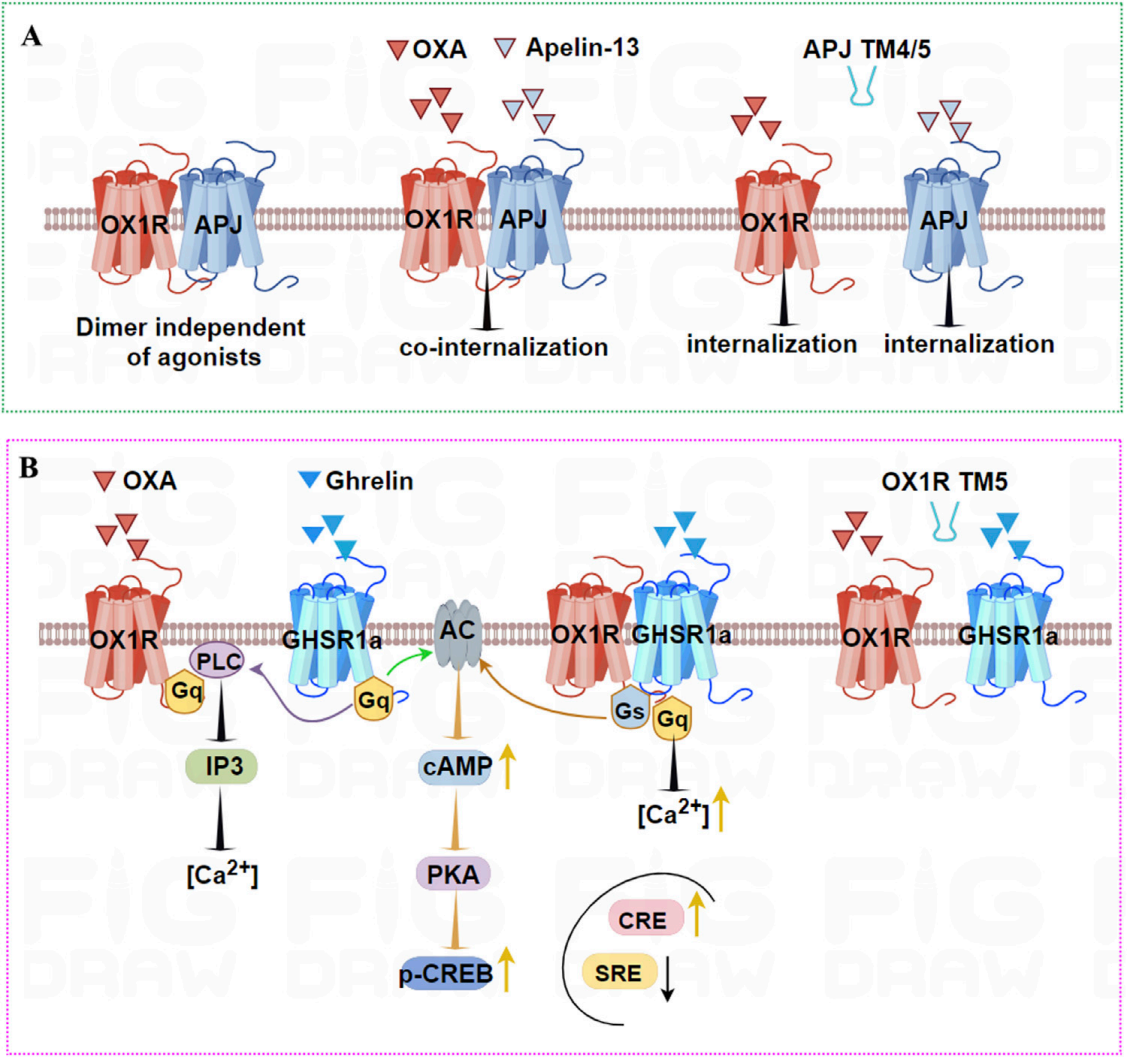
OX1R binding to APJ or GHSR1a results in heterodimers that can be disrupted by TM peptides. **(A)** OX1R and APJ can form heterodimers independent of agonists. Using OXA or Apelin-13 causes co-internalization of the two receptors. In addition, TM4/TM5 of APJ leads to separation of the heterodimer. **(B)** OX1R and GHSR1a can form heterodimers that may elevate cAMP levels and induce higher intracellular [Ca2+] levels upon ghrelin stimulation. Ghrelin stimulation increases CRE activity and decreases SRE activity, but OXA treatment has no effect on CRE activity. TM5 of OX1R blocks the formation of the heterodimer interface.

Co-IP and BRET revealed that OX1R and APJ form a heterodimer in HEK293T cells (Wan et al., 2020). Furthermore, ligand binding does not affect the BRET ratio or the concentration gradient of APJ-OX1R dimerization, indicating that OX1R and APJ constitutively form a heterodimer. The OX1R-APJ dimer internalize subject to binding either apelin-13 or OXA. Interestingly, incubation with dual agonists can lead to even greater internalization, which was disturbed by transmembrane (TM) helices 4 and 5 of APJ *in vitro*. These findings strongly indicate that OX1R dimerizes with APJ, with TM 4 and 5 of APJ serving as a primary interface for this interaction, providing powerful evidence for the benefits of studying the interaction interfaces of GPCR heterodimerization (Figure 4A), which can be severed as a potential pharmacological target.

#### 4.2.4 OX1R and GHSR1a heterodimerization

GHSR1a, a member of the GPCR family, is highly expressed in the hippocampus, hypothalamus, VTA, and substantia nigra pars compacta (SNpc). GHSR1a is a functional receptor of ghrelin, a brain-gut peptide composed of 28 amino acids. The ghrelin/GHSR1a system couples to Gα_q_ to activate PLC, release IP_3_, and mobilize Ca^2+^, thereby regulating various biological activities including the release of GH, learning and memory, and reward and feeding (Guan et al., 1997; Holst et al., 2003). OX1R and GHSR1a have many similar distributions and functions in the CNS, suggesting that OX1R/GHSR1a dimerization may modify their original functions, which plays an important role in the ghrelin and orexin system.

The novel OX1R/GHSR1a heterodimer was discovered using BRET, FRET, Co-IP, and PLA (Xue et al., 2018). TM5 of OX1R is involved in the formation of the heterodimer interface. OX1R/GHSR1a may enhance cAMP levels in a dose-dependent manner upon stimulation by ghrelin, but not OXA, suggesting that OX1R/GHSR1a may strengthen Gα_s_ coupling following ghrelin stimulation. Ghrelin stimulates OX1R/GHSR1a heterodimer cells to increase CRE activity and decrease SRE activity. However, treatment of OX1R/GHSR1a cells with OXA had no effect on CRE activity. The results of Ca^2+^ mobilization analysis indicated that GHSR1a activation leads to higher [Ca^2+^] levels than OX1R (Figure 4B). Furthermore, BiFC-BRET revealed that OX1R/GHSR1a heterodimers mainly activate Gα_s_ but not Gα_i_ and Gα_q_. These results indicate that after the formation of heterodimers between OX1R and GHSR1a, ghrelin biases toward Gα_s_ signaling, promoting the upregulation of the Gα_s_-cAMP-cAMP response element signaling pathway and the increase in neuroblastoma cell proliferation, possibly exerting a neuroprotective effect.

#### 4.2.5 OX1R and CCK1R heterodimerization

Cholecystokinin (CCK), the first gastrointestinal hormone discovered, is a 33 amino acid peptide (CCK-33). Cholecystokinin receptors (CCKRs) belong to the GPCR superfamily and are divided into two types (CCK1R and CCK2R). CCK1R and OX1R are mainly distributed in cerebral cortex and hypothalamus. Functionally, CCK1R and OX1R dimers are related to the regulation of appetite in the body, and they have antagonistic effects on each other (Noble et al., 1999). These two receptors bind to the Gα_q_ subunit, altering downstream signaling. Both OX1R and CCK1R are linked to the migration of human colon cancer cells. The functional activities of OX1R and CCK1R in the migration of colon cancer are mediated through their interactions (Bai et al., 2017).

**FIGURE 5:**
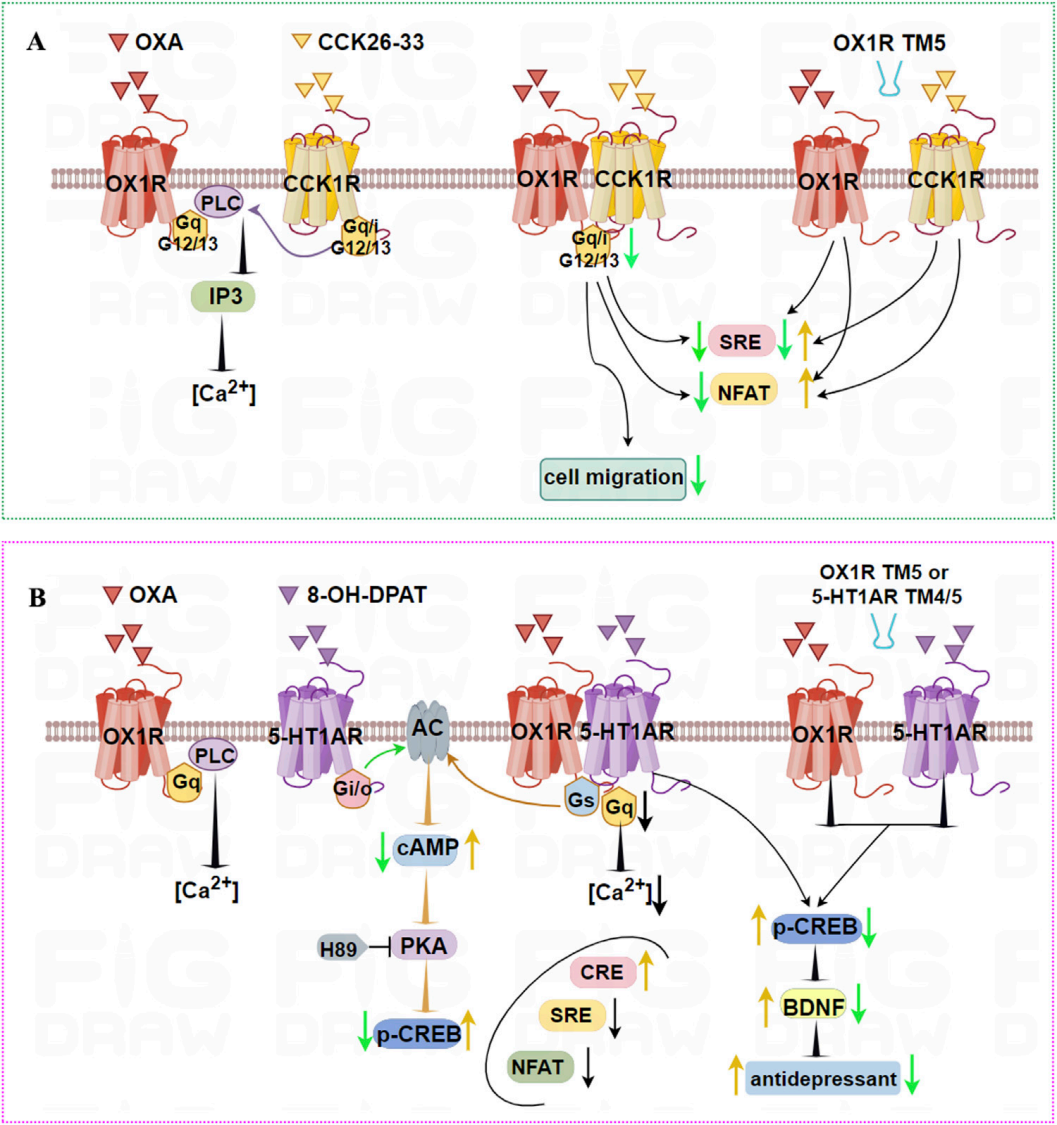
Illustration of the signaling pathways induced by dimerization of OX1R with CCK1R or 5-HT1AR, and TM peptides. **(A)** OX1R and CCK1R can form stable heterodimers that inhibit activation of Gα_q_, Gα_i_, Gα_12_, and Gα_13_, and migration of HT-29 human colon cancer cells. TM5 of OX1R impairs the heterodimerization of OX1R and CCK1R, resulting in higher NFAT activity when treated with OXA or CCK 26–33, low SRE activity following OXA treatment, but higher SRE activity in response to CCK 26–33 treatment. **(B)** OX1R with 5-HT1AR can form constitutive heterodimers. When co-stimulated by OXA and 8-OH-DPAT, the ability of OX1R/5-HT1AR to bind Gα_q_ is weaker and the intracellular [Ca^2+^] concentration is decreased. The ability of OX1R/5-HT1AR to bind Gα_s_ is significantly higher, leading to increased cAMP levels and p-CREB. Similarly, CRE levels are upregulated, but NFAT and SRE signal factors are significantly downregulated. The antidepressant effects of heterodimers may be mediated by upregulated p-CREB and BDNF levels. However, TM4/TM5 peptides interface with OX1R/5-HT1AR heterodimers, aggravating their depressive status.

OX1R and CCK1R form stable heterodimers that are both structurally constitutive and ligand-induced, and TM5 of OX1R blocks heterodimer formation. Endogenous OX1R and CCK1R heterodimerize in HT-29 colon cancer cells based on *in situ* PLA technology. In general, OX1R couples to Gα_q_, Gα_12_, and Gα_13_, whereas CCK1R couples to Gα_q_, Gα_i_, Gα_12_, and Gα_13_. However, OX1R and CCK1R heterodimers inhibit the activation of Gα_q_, Gα_i_, Gα_12_, and Gα_13_ compared with stimulation by OXA or CCK alone, but do not alter GPCR interactions with β-arrestins (Figure 5A). Moreover, OX1R/CCK1R heterodimers inhibit the migration of HT-29 cells stimulated by dual agonists, suggesting that OX1R/CCK1R heterodimerization plays an anti-migratory role in human colon cancer cells, providing new targets for cell anti-migration in the treatment of cancer.

#### 4.2.6 OX1R/OX2R and 5-HT1AR heterodimerization

Serotonin 1A receptor (5-HT1AR) is widely distributed in the hippocampus, hypothalamus, cortex, striatum, and midbrain regions, and especially abundant in the hippocampus and cortex. Binding of 5-HT to 5-HT1AR mainly activates the Gα_i/o_ subtype, inhibits AC activation, downregulates cAMP levels, and modulates a number of processes associated with mood, reward, aggression, appetite, and psychiatric diseases in the CNS (Howick et al., 2017; Maroteaux et al., 2019). There are some overlaps between the roles and expression profiles of OX1R and 5-HT1AR in the CNS. For example, HCRT fibers project toward both 5-HT and GABAergic neurons in the DRN. Interestingly, 5-HT directly hyperpolarizes orexin neurons by enhancing potassium conductance, and this reaction can be suppressed by a specific antagonist of 5-HT1AR (Muraki et al., 2004).

OX1R and 5-HT1AR form constitutive heterodimers that mediate novel G protein-dependent signaling without affecting recruitment of β-arrestin (Zhang R. et al., 2022). When co-stimulated by OXA and 8-OH-DPAT, the ability of OX1R/5-HT1AR to bind the Gα_q_ subunit was weaker than for OX1R stimulated by alone OXA. Meanwhile, the ability of OX1R/5-HT1AR to bond the Gα_s_ subunit was significantly higher than for OX1R stimulated by OXA and 5-HT1AR stimulated by 8-OH-DPAT, respectively. OX1R/5-HT1AR heterodimers significantly lowered the [Ca^2+^], but cAMP levels were markedly increased. Similarly, CRE levels were upregulated, but NFAT and SRE signal factors were significantly downregulated. Using PKA inhibitor H89 and PLC inhibitor U73122, it was found that OX1R/5-HT1AR dimers affected CREB phosphorylation mainly via the cAMP/PKA pathway rather than the PLC/PKC pathway (Figure 5B). These results indicate that heterodimers of OX1R and 5-HT1AR preferentially bind Gα_s_. Using the PLA method, OX1R/5-HT1AR dimer levels in CUMS rats were significantly decreased compared with the control group, while OXA or 8-OH-DPAT significantly increased levels of 5-HT1AR/OX1R dimers. TM4/TM5 peptides aggravate the depressive status and decrease the number of endogenous 5-HT1AR/OX1R heterodimers in CUMS rats. The antidepressant effects were mediated by upregulated BDNF levels and increased phosphorylated CREB content. This indicates that OX1R/5-HT1AR heterodimers affect the pathological process of depression. Peptides including TMs of the 5-HT1AR/OX1R heterodimer interface are candidates for the development of compounds with fast-acting antidepressant-like effects.

**FIGURE 6:**
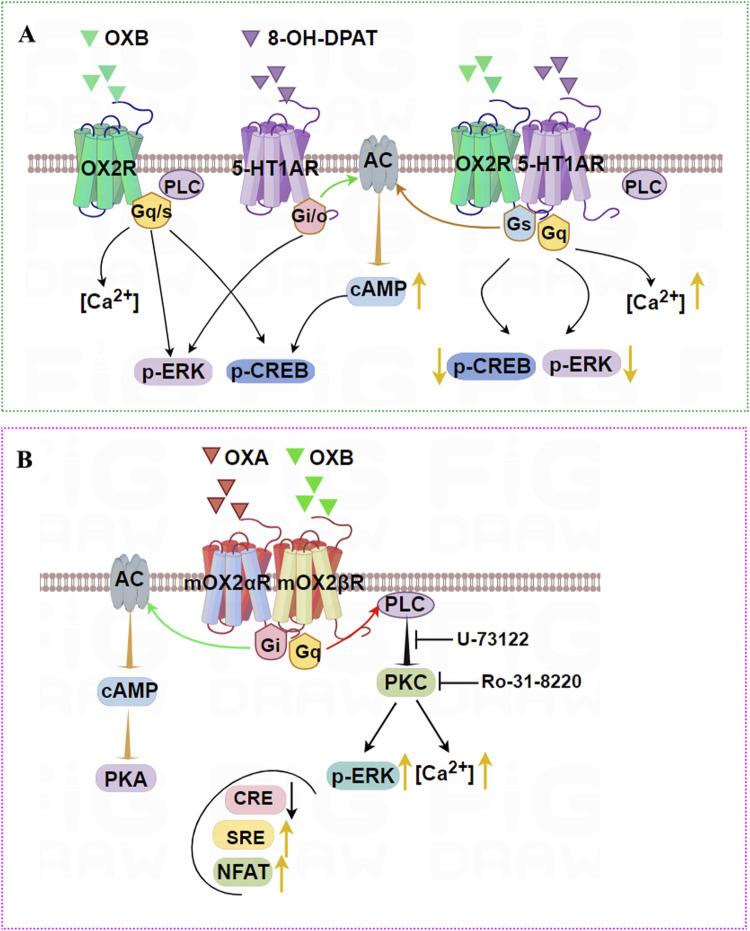
Illustration of the signaling pathways induced by OX2R heterodimers. **(A)** OX2R and 5-HT1AR can form heterodimers that are stimulated by OXB and 8-OHDPAT, leading to an obvious increase in cellular cAMP and intracellular [Ca^2+^] compared with single agonist stimulation. By contrast, OX2R and 5-HT1AR heterodimers robustly decrease levels of p-ERK and p-CREB compared with OX2R or 5-HT1AR-transfected groups. **(B)** mOX2αR and mOX2βR can form heterodimers. After OXA/OXB stimulation, dimerization causes a significant activation of p-ERK_1/2_ and increases intracellular [Ca2+] via PKC activation. In addition, dimerization enhances the activity of NFAT and SRE activity depending upon Gα_q_-PLC, and decreases CRE activity depending on PKA activity when OXA or OXB are added.

Another report in our lab firstly demonstrated that OX2R and 5-HT1AR also form heterodimers in HEK293T cells and in rat hippocampus (Wang et al., 2019). These heterodimers were stimulated with 8-OHDPAT and OXB, leading to an obvious increase in cellular cAMP and calcium influx compared with single or no agonist stimulation. By contrast, OX2R and 5-HT1AR heterodimers markedly decreased levels of p-ERK and p-CREB compared with OX2R or 5-HT1AR transfection groups. These results not only unraveled the formation of 5-HT1AR and OX2R heterodimer but also suggested that the heterodimer affected the downstream signaling pathway, which will provide new insights into the function of the OX2R or 5-HT1AR (Figure 6A) in the brain.

#### 4.2.7 Heterodimerization of mouse OX2R variants

mOX2αR and mOX2βR are two alternative C-terminus splice variants of the mouse Orexin receptor. The main structural difference lies in their C-terminal regions, which result in distinct functional properties. mOX2αR is the full-length receptor, while mOX2βR likely excludes specific exons, leading to a truncated protein. mOX2αR likely couples to Gα_q_, whereas mOX2βR shows biased signaling or coupling to additional G-proteins (e.g., Gi/o), altering downstream effects like cAMP modulation. Although there were no differences in affinity between OXA and OXB for mOX2αR and mOX2βR, interaction between OXB and mOX2βR appeared to achieve a higher maximum IP response than mOX2αR (Chen and Randeva, 2004).

Dimerization of mOX2αR and mOX2βR caused significant activation of p-ERK_1/2_ in both time- and dose-dependent manners (Wang et al., 2014). Furthermore, after OXA/OXB stimulation, heterodimerization dramatically increased [Ca^2+^] through PKC activation. In addition, the increased SRE and decreased CRE activity depended on PKC activity, and PKA activity was added to HEK293-mOX2αR/mOX2βR cells. Dimerization also enhanced the activity of NFAT-RE via a mechanism dependent on Gα_q_/PLC pathways (Figure 6B). In conclusion, we reported that mouse OX2αR forms a heterodimer with OX2βR. This heterodimerization process drives activation PKA and PKC-signaling pathways upon activation by Orexins. Furthermore, intracellular Ca^2+^ via activation of Gα_q_ was elevated rapidly after stimulation by corresponding agonists, and cell proliferation was also significantly increased.

#### 4.2.8 OX1R/OX2R and GRP103 heterodimerization

It has long been suspected that there may be some involvement of the orexigenic system in AD. For example, association between mean Aβ 42 CSF levels and OXA levels reportedly indicates a relationship between AD pathology and orexin disturbance (Slats et al., 2012). QRFP, a ligand of GPR103, exerts similar orexigenic activity including energy homeostasis and feeding behavior. The high sequence identity between GPR103 and the orexin receptors (OX1R, OX2R), along with their overlapping expression in the PVN and VMH, combined with their overlapping functional domains, assumed that there may be some association of the OX/OXR and QRFP/GRP103 systems and in AD (Jiang et al., 2003).

The expression, distribution, and signaling characteristics of OXR and GRP103 in the AD brain have been investigated (Davies et al., 2015). Expression of OX1R, OX2R, and GPR103 in early onset familial AD (EOFAD) and late onset non-familial AD (LOAD) patients was significantly reduced compared with healthy controls. There was a positive correlation between OX1R, OX2R, and GPR103 in EOFAD. However, for LOAD, the only positive correlation was between OX2R and GPR103. BRET and FRET results showed that OX1R and GPR103 or OX2R and GPR103 could form constitutive and induced heterodimers. Moreover, all three peptides could increase p-ERK_1/2_ levels, which was abolished by selective OX1R antagonist SB-334867 and OX2R antagonist TCSOX 229. Moreover, OXR antagonists also blocked QRFP-induced p-ERK_1/2_ in differentiated cells, indicative of a crosstalk mechanism between OXRs and GPR103. In this study, given that dimerization of GPCRs is critical for receptor function, including specificity in signal transduction, it is first demonstrated that GPR103 forms a functional heterodimer with OXRs, thereby playing a potential neuroprotective role. Pharmacological interventions targeting the orexin system were an attractive avenue to discover new treatments for diseases such as AD and improve neuroprotective signaling pathways.

Based on the above research, receptor dimerization exhibits significant bias in the selection of downstream signaling molecules. This bias leads to different physiological effects, thereby affecting the functions and fates of cells. In pharmacology, understanding and utilizing biased signaling pathways is of great significance for the development of novel drugs. By precisely adjusting the bias of signaling pathways, more efficient and specific drugs can be developed.

Although there is ample evidence in recombinant cell systems that OXR can form dimers, these dimerization processes may affect the ligand binding characteristics, signal transduction pathways and intracellular transport of receptors, thereby regulating their functions. However, the evidence verified *in vivo* remains limited and faces many technical challenges. For instance, common techniques like BRET and FRET are powerful for detecting proximity in live cells but are limited by their resolution; they cannot distinguish direct dimerization from mere proximity within a large protein complex. Furthermore, bimolecular fluorescence complementation (BiFC) can demonstrate direct interaction but is prone to false positives from the irreversible and spontaneous reassembly of fluorescent protein fragments. Most of the existing *in vivo* studies are indirect and lack evidence for direct detection of endogenous receptor dimerization. Future research should pay more attention to the changes of receptor dimerization under disease conditions, develop drugs targeting receptor dimerization, and integrate multidisciplinary approaches to comprehensively understand this complex phenomenon.

## 5 Neuropharmacology (OXR antagonists)

Currently, most OXR antagonists block the effects of promoting and maintaining wakefulness to treat insomnia. They are also expected to become drug targets for treating obesity, addiction, and neurological or psychiatric disorders. According to their binding affinity, OXR antagonists were classified into selective OX1R antagonists (SORA1s), selective OX2R antagonists (SORA2s), and DORAs.

### 5.1 SORA1s

#### 5.1.1 SB-334867

The first SORA1 developed in 2001 was SB-334867, which has 50-fold higher selectivity for OX1R than OX2R. It can induce small increases in REM and non-REM (NREM) sleep (Morairty et al., 2012), but it cannot increase total sleep duration, nor can it weaken wakefulness or reduce sleep latency time (Staples and Cornish, 2014).

In addition, SB-334867 has the potential to regulate appetite and addiction. SB334867 can inhibit eating behavior after fasting and injection of OXA. SB-334867 injected into the VTA not only reduced self-administration of cocaine and blocked cue-induced cocaine relapse (Aston-Jones et al., 2009; Borgland et al., 2006), but also inhibited the formation of conditioned place preference (CPP) for morphine. Microinjection of SB-334867 into the medial nucleus accumbens shell significantly reduced alcohol intake in C57BL/6J mice, especially in heavily alcohol-consuming mice (Lei et al., 2019).

SB-334867, mainly at the dose of 10 μg/rat, decreased the median of seizure stages, prolonged the latency and reversed the PTZ-induced anxiety-like behaviors, suggested that pharmacological blockade of the OX1 receptor is a potential target in the treatment of seizure and concomitant anxiety disorders (Jaz et al., 2017).

#### 5.1.2 Nivasorexant (ACT-539313)

Nivasorexant was the first SORA1 to enter clinical development and completed a first proof of concept phase II clinical trial for binge-eating disorder in 2022 (Kaufmann et al., 2021). In phase II study, Nivasorexant was well tolerated in adults with binge-eating disorder (McElroy et al., 2023). Nivasorexant reduced binge-like eating in a dose-dependent manner, and its efficacy was maintained under chronic administration and more frequent stress exposure (Steiner et al., 2024).

Preclinical studies have indeed shown that OX1R antagonists may be effective in reducing binge eating in humans, but Nivasorexant did not achieve the expected therapeutic effect in a phase 2 clinical trial (https://clinicaltrials.gov/) Possible reasons could be that the dosage used in the trial may not have reached the optimal treatment window or that the etiology and symptoms of binge-eating disorder patients are diverse, which may have affected the overall response of the drug. According to publicly available trial records, Nivasorexant has good safety data, with common adverse reactions ranging from mild to moderate (such as headache and nausea) and no reported serious side effects. This provides a foundation for its subsequent research.

#### 5.1.3 CVN766

CVN766 is a novel and highly selective SORA1 currently being evaluated in clinical trials for schizophrenia overactivation of OX1R is associated with positive symptoms of schizophrenia (such as hallucinations, delusions) and cognitive impairment. CVN766 may regulate dopaminergic and glutamatergic pathways by selectively antagonizing OX1R, thereby improving symptoms. Compared with DORAs or other non-selective drugs, CVN766’s extremely selective response to OX1R may reduce off target effects and improve safety. However, CVN766 may be in the early stages of clinical trials (such as phase I or II), and further validation of its efficacy and safety is needed (Glen et al., 2024).

### 5.2 SORA2s

#### 5.2.1 EMPA

The first SORA2, EMPA, has 900-fold stronger selectivity for OX2R than OX1R. EMPA can selectively increase NREM but has no effect on REM sleep or sleep onset time (Malherbe et al., 2009). It can also reduce spontaneous movement disorders and disturbances caused by OX2R, but compared with other SORA2s, its sleep-promoting activity is lower.

#### 5.2.2 Seltorexant (JNJ-42847922)

Seltorexant can dose-dependently reduce the latency and prolong the duration of NREM but has very little effect on REM sleep. Repeated administration of JNJ-42847922 (30 mg/kg) within 7 days can maintain a decrease in latency to sleep and an increase in sleep duration, and sleep parameters return to baseline levels after stopping the medication (Bonaventure et al., 2015; Recourt et al., 2019). Following treatment with Seltorexant, patients with severe depression and insomnia showed significant improvements in both mood and sleep quality (Recourt et al., 2019), and this compound was identified as a clinical candidate drug for treating depression.

### 5.3 DORAs

It is speculated that inhibiting two OXRs simultaneously is more effective at inducing sleep than inhibiting any one OXR alone and selectively inhibiting a single OXR does not appear to show great potential for treating insomnia. Subsequent research turned this speculation into a reasonable inference, designing and synthesizing most DORAs. Drugs approved by the US Food and Drug Administration (USFDA) for treating insomnia include Suvorexant, Lemborexant, and Daridorexant.

#### 5.3.1 Suvorexant

Suvorexant (Belsomra®) was developed by Merck MSD and was the first DORA approved by the USFDA for marketing. This drug was launched in the United States in August 2014 for the treatment of insomnia caused by difficulty falling asleep and/or maintaining sleep.

In clinical trials, when healthy subjects took the drug, Suvorexant significantly reduced the latency to persistent sleep, shortened the awakening time after sleep onset, and improved sleep quality (Sun et al., 2013). When patients with primary insomnia received Suvorexant, it exhibited obvious dose-related improvements vs. controls on the coprimary end points of sleep efficiency, increasing REM and therefore overall sleep duration, without adverse reactions such as residual effects the following day or rebound insomnia (Herring et al., 2012). In a clinical trial for vasomotor symptom (VMS)-associated insomnia disorder in middle-aged women, Suvorexant was found to efficaciously reduce nighttime VMS, providing a new therapeutic option for middle-aged women with VMS-related chronic insomnia (Rahman et al., 2022).

Suvorexant has shown good clinical efficacy in the elderly, improving total sleep duration in patients who may suffer from AD dementia and insomnia. It also displayed good tolerance and only mild adverse reactions, indicating a positive effect on AD patients with insomnia (Herring et al., 2020). In another study on cognitively unimpaired AD participants aged 45–65 years, Suvorexant acutely decreased Tau phosphorylation and amyloid-β concentrations in the CNS (Lucey et al., 2023).

In type 2 diabetes patients, Suvorexant improved sleep quality and obesity-related parameters, including blood glucose control, consistent with data obtained from preclinical experiments on diabetes model mice (Tsuneki et al., 2016). Suchting et al. (2020) used Suvorexant for clinical treatment of cocaine dependence, marking the first clinical study on addiction-related use. Suvorexant improved sleep, reduced physiological responses to acute stress, and decreased patient craving for cocaine (Suchting et al., 2020). Prajapati et al. demonstrates that Suvolesen may alleviate post-traumatic stress disorder (PTSD) symptoms by regulating orexin, serotonin and the neuroendocrine system, and considering orexin A as a neurochemical marker of PTSD (Prajapati and Krishnamurthy, 2021). They also proved that suvorexant can significantly alleviate PTSD-like symptoms and improved mitochondrial dynamics. In addition, suvorexant can significantly mitigate mTOR and MTFP-1 activation, indicating that the pharmacological effects of suvorexant involve the mTOR pathway in mitochondrial biogenesis (Prajapati et al., 2024).

Although Suvorexant is relatively safe, it is still necessary to consider associated adverse effects such as dizziness, headache, somnolence, and cognitive impairment.

#### 5.3.2 Lemborexant

Lemborexant (Dayvigo) was the second oral DORA to be approved, in the United States in December 2019 and in Japan in January 2020, for the treatment of insomnia in adults with sleep onset and sleep maintenance difficulties.

Lemborexant and Suvorexant both function as reversible competitive antagonists at the orexin receptors, consistent with the mechanism of action of DORAs. However, Lemborexant is more potent at the OXR2 receptor than Suvorexant. Its inhibitory effect is significantly better on OX2R than OX1R, and it has higher selectivity, thus providing stronger anti-insomnia effects. Habiba et al. (2023) evaluated the efficacy of Lemborexant therapy and reported a significant dose-dependent improvement in sleep onset latency, sleep efficacy and quality, total sleep duration, and morning alertness (Habiba et al., 2023).

Sleep disorders are associated with an increased risk of type 2 diabetes (Tsuneki et al., 2023). Administration of Lemberexant can significantly increase the total duration of NREM and REM sleep in diabetes mice, increase the duration of NREM sleep, REM sleep, and awake seizures, and improve glucose tolerance, resulting in an increase in exercise activity of diabetes mice during the dark period.

Studies have shown that compared with placebo, Lemborexant does not affect daytime function the next day, and it can effectively reduce adverse reactions such as next day residual effects (Moline et al., 2021; Ueno et al., 2021). Although Lemborexant does have mild side effects, its clinical safety and efficacy make it a promising insomnia medication.

#### 5.3.3 Daridorexant

Daridorexant (Quviviq), developed by Idorsia, was approved by the USFDA on 10 January 2022, becoming the third USFDA-approved DORA drug for the treatment of adult insomnia patients characterized by difficulty falling asleep and/or maintaining sleep. Daridorexant has the ability to equivalently antagonize OX1R and OX2R (Markham, 2022), effectively crossing the BBB and interacting with OXRs in the brain, thereby regulating the processes of sleep and wakefulness. It can specifically block the binding of arousal-promoting neuropeptides OXA and OXB to OX1R/OX2R (Treiber et al., 2017). Therefore, Daridorexant can inhibit the awakening drive of animals and humans and promote sleep.

A series of clinical trials showed that Daridorexant has significant therapeutic effects on insomnia patients, as it can shorten the latency period to sleep, prolong total sleep duration, and reduce wakefulness after falling asleep (Dos Santos and da Silva, 2022; Dutta et al., 2023). Daridorexant can also reduce the frequency of waking up at night and improve the quality of sleep throughout the night. Additionally, it has little impact on daytime function the next day, is well tolerated, and has no residual effects the next morning (Uchimura et al., 2024). Daridorexant has a good pharmacokinetic curve *in vivo* that aligns well with the natural sleep cycle of the human body. Treatment with Daridorexant for up to 12 months, and sustained improvements in sleep and daytime functioning, support its use for long-term treatment of insomnia (Kunz et al., 2023). Overall, it is safe and well tolerated.

### 5.4 TM peptides

GPCRs generally contain 7 TM domains that govern the occurrence and stability of dimerization (Chen et al., 2021). TM peptides designed from TM domains interact with GPCRs, not only blocking the formation of dimers in many cases, but also inhibiting ligand-induced signaling, ultimately affecting their function.

TM domains are a current hot topic in studying GPCR interactions, including TM1-2, TM3, TM4-5, and TM6-7. TM1-2 mainly participates in homodimerization, as reported for neurotensin 1 receptor (NTS1R) homodimers (Casciari et al., 2008). Our lab demonstrated that TM1, 2, 3, and 4 of APJ form the homodimer interface (Cai et al., 2017). TM4-5 can directly participate in the heterodimerization of A2A adenosine receptor (A2AR) and D2 dopamine receptor (D2R). Peptides corresponding to TM-4 and TM-5 of A2AR disrupted heterodimeric interactions between A2AR and D2R, and blocked the allosteric effect of A2AR activation on D2R agonist binding (Borroto-Escuela et al., 2018). TM peptides corresponding to TM6/TM7 of CCK1R suppressed receptor dimerization without changing their functions (Harikumar et al., 2006). Our lab also found that TM4-5 of APJ is the dimerization interface of APJ and OX1R. This discovery adds new evidence that TM4-5 is an important interface in GPCR isomerization (Wan et al., 2020).

Studies suggest that TM peptides may have therapeutic potential because they interfere with dimerization and further affect receptor function, which is of great significance for the development of novel drugs. The TM5/TM6 peptide derived from CB1R alters the structure of the CB1R-5HT2AR heterodimer, avoiding cognitive impairment in animals while retaining analgesic effects. Based on the TM5 and TM6 sequences, orally active 16-residue peptide with analgesic effects was ultimately identified as a candidate drug (Gallo et al., 2021). Our lab examined the antidepressant-like effects of TM peptides *in vivo*. After injection of one of 3 TM peptides (5-HT1AR-TM4, 5-HT1AR-TM5, and OX1RTM5) into the lateral ventricle of CUMS rats, specific depression-related functions were reverted via inhibiting the interface of the 5-HT1AR/OX1R heterodimer (Zhang R. et al., 2022). Therefore, targeting the GPCR interface using TM peptides will be used to develop highly selective therapeutic drugs.

In an article that we published in Drug Discovery Today, we first proposed that small-molecules composed of GPCR TM peptides interfere with or promote receptor dimerization to achieve therapeutic goals (Cai et al., 2023). Small molecules or reagents, such as monovalent antibodies or transmembrane peptides, that target the interfaces of GPCR homodimers and heterodimers can serve not only as crucial tools for elucidating the physiological significance of these complexes but also as “lead compounds” for developing highly selective drugs to treat a variety of diseases. Future research should validate dimer-specific signaling *in vivo* and explore TM peptide-based interventions to selectively disrupt or enhance receptor complexes, potentially revolutionizing treatment strategies for insomnia, depression, addiction, and neurodegenerative diseases.

## 6 Conclusion and future directions

As targets of numerous drugs, GPCRs have attracted widespread attention for their structural and functional properties, signaling pathways, and antagonists. This review focuses on the compositional characteristics, signal transduction, dimerization, and antagonists of OXRs, which can form heterodimers that behave differently to monomers in signaling pathways, conferring OXRs with more functions. We explore signaling resulting from functional dimerization of OXRs with other GPCRs and associated physiological processes and related diseases. The biased signals induced by dimerization of OXRs could be more effectively utilized for drug development to enhance pharmacological treatment of various diseases including insomnia and depression.

Notably, while emerging studies have suggested that dimerization of OXRs and biased signal transduction may contribute to certain biological processes and disease pathogenesis, recent evidence indicates this regulatory mechanism requires further validation and may exhibit context-dependent characteristics. Animal experiments are therefore needed to confirm receptor dimerization and its impact on biased signal transduction *in vivo*, potentially providing new insights into orexin/receptor systems and associated diseases. Furthermore, considering the critical regulatory roles of these systems in health and disease, there is an urgent need for physiological research in both healthy and diseased tissues.

The emergence of DORAs has to some extent solved a series of problems associated with mainstream hypnotic drugs, allowing patients a more natural sleep experience. However, as regulators of multiple physiological activities in the body, when using OXR antagonists it is worth considering whether they exert multiple effects simultaneously or disrupt a certain physiological process and cause adverse reactions. Additionally, the safety of using DORAs requires further investigation.

## Abbreviations

OXRs, Orexin receptors; GPCRs, G protein-coupled receptors; HCRTR, Hypocretin receptor; OXA/OXB, orexin A/orexin B; PPO, prepro-orexin; CNS, central nervous system; IRI, cerebral ischemia-reperfusion injury; ERS, endoplasmic reticulum stress; CUMS, chronic unpredictable mild stress; LH, lateral hypothalamus; VMH, ventromedial hypothalamus; CA, hippocampus; DR, dorsal raphe; LC, locus coeruleus; PVN, paraventricular nucleus; PPT, pontine tegmental nucleus; LDT, dorsal lateral tegmental nucleus; VTA, ventral tegmental area; CHO, Chinese hamster ovary; ER, endoplasmic reticulum; NPY, neuropeptide Y; TRPC, transient receptor potential canonical; Dynlt, Dynein light chain Tctex-type; MRAP, Melanocortin Receptor Accessory Protein; ITIMs, immunoreceptor tyrosine-based inhibitory motifs; BRET, bioluminescence resonance energy transfer; KOR kappa opioid receptor; CB1R, cannabinoid receptor 1; APJ, apelin receptor; GHSR1a, growth hormone secretagogue receptor 1a; CCKR, cholecystokinin receptor; 5-HT1AR, 5-hydroxytryptamine 1A receptor; GPR103, G protein-coupled receptor 103; Dyn, dynorphin; FRET, fluorescence resonance energy transfer; CRE, cAMP-response element; SRE, serum response element; NFAT, nuclear factor of activated T-cells; TM, transmembrane; DORA, orexin receptor antagonist; SON, supraoptic nucleus; SNpc, substantia nigra pars compacta; CCK, Cholecystokinin; EOFAD, early onset familial AD; LOAD, late onset non-familial AD; SORA1s, selective OX1R antagonists; SORA2s, selective OX2R antagonists; REM, rapid eye movement; NREM, non-REM; CPP, conditioned place preference; USFDA, US Food and Drug Administration; VMS, vasomotor symptom; NTS1R, neurotensin 1 receptor; A2AR, A2A adenosine receptor; D2R, D2 dopamine receptor.
